# Adult-Onset Alagille Syndrome Presenting With Recurrent Cholestasis: A Rare Genetic Diagnosis Confirmed by Whole Exome Sequencing

**DOI:** 10.7759/cureus.114279

**Published:** 2026-08-10

**Authors:** Sayali D Kolse, Shilpa A Deoke, Arvind Agrawal, Krunal Domki

**Affiliations:** 1 Internal Medicine, NKP Salve Institute of Medical Sciences and Research Centre and Lata Mangeshkar Hospital, Nagpur, IND

**Keywords:** adult-onset cholestasis, alagille syndrome, jag1 mutation, recurrent cholestasis, whole exome sequencing (wes)

## Abstract

Alagille syndrome, also known as arteriohepatic dysplasia, is a multisystem genetic disorder inherited in an autosomal dominant pattern. It is primarily caused by mutations in the Jagged canonical Notch ligand 1 (JAG1) gene and is classically diagnosed in childhood with cholestasis due to bile duct paucity. Adult presentation is rare owing to its incomplete penetrance and milder phenotypes.

We report a case of a 33-year-old male with recurrent cholestasis and no prior history of childhood jaundice. Common acquired etiologies were excluded, and comprehensive evaluation revealed a JAG1 mutation by whole exome sequencing (WES). Multiorgan screening showed no extrahepatic involvement. Treatment with ursodeoxycholic acid (UDCA) and supportive therapy led to clinical and biochemical resolution. This case emphasizes the significance of considering rare genetic disorders in adults with unexplained recurrent cholestasis and highlights the value of molecular diagnostics such as whole exome sequencing in facilitating timely diagnosis.

## Introduction

Alagille syndrome is inherited in an autosomal dominant manner, predominantly caused by heterozygous pathogenic variants in the JAG1 (95% of cases) or NOTCH2 genes, leading to disruption of Notch signaling and giving rise to characteristic bile duct paucity and multiorgan anomalies. Although deletions involving the short arm of chromosome 20 (20p12) that encompass JAG1 can occur, they account for only a minority of cases; point mutations and small insertions/deletions are far more common [1]. This syndrome was first identified by Dr. Daniel Alagille, a French pediatric hepatologist, in 1969, based on a series of children with intrahepatic bile duct paucity and extrahepatic features, such as cardiac defects, vertebral anomalies, renal dysplasia, and characteristic facial dysmorphism [2].

Due to variable expressivity and incomplete penetrance, this syndrome occurs in approximately one in 30,000-50,000 live births, although the true prevalence may be underestimated [3]. With improved survival, approximately 90% of children with Alagille syndrome with or without cholestasis now survive beyond 18 years of age, making adult presentations increasingly recognized.

However, there is a well-recognized lack of genotype-phenotype correlation in Alagille syndrome, which has been reported in several studies on Alagille syndrome presenting in adulthood [3]. Despite carrying the same JAG1 pathogenic variant, patients often exhibit marked variability in disease severity and organ involvement, often presenting in adulthood with isolated or recurrent cholestasis with or without syndromic features, making the diagnosis particularly challenging [4]. Recurrent cholestasis in adulthood frequently poses a diagnostic challenge, as clinicians typically prioritize common acquired etiologies, such as viral hepatitis, autoimmune liver disease, or drug-induced liver injury, before considering a genetic etiology. In the era of molecular genetic testing, whole exome sequencing (WES) has emerged as a valuable diagnostic tool for confirming rare inherited disorders with atypical presentations [5].

Here, we describe a case of Alagille syndrome in a patient with recurrent cholestatic episodes who presented in adulthood and was diagnosed by whole exome sequencing (WES), highlighting the role of genetic evaluation in patients with unexplained intrahepatic cholestasis.

## Case presentation

A 33-year-old male presented with complaints of yellowish discoloration of the eyes and body, along with pruritus for 10 days. He denied a history of fever, abdominal pain, vomiting, weight loss, hematemesis, melena, or any autoimmune disease. He had a history of occasional alcohol intake, which he had abstained from for the last four years, and also reported no recent exposure to drugs or toxins. Two years prior, he experienced a similar self-resolving episode for which he was admitted and diagnosed as probable drug-induced liver injury based on history of herbal medication use (details of the specific drug were not available in the records). The episode resolved spontaneously without any specific long-term therapy. Patient, however, gave no history of childhood jaundice. Family history was notable as his younger sibling had a history of jaundice and was deceased (details were unknown), but had no dysmorphic features as informed by the patient. The clinical timeline of events in our patient is summarized in Table 1.

**Table 1:** Key events in clinical course of the patient. USG: ultrasonography; UDCA: ursodeoxycholic acid; INR: international normalized ratio; BRIC: benign recurrent intrahepatic cholestasis; PFIC: progressive familial intrahepatic cholestasis; WES: whole exome sequencing; LFT: liver function test; HCC: hepatocellular carcinoma; GGT: gamma-glutamyl transferase

Timeline	Event
2 years prior	First episode of jaundice and pruritus, diagnosed as probable drug-induced liver injury based on recent medication use and exclusion of common acquired causes; resolved spontaneously
Day 0	10 days history of jaundice and pruritus
Day 1-3	Lab evaluation, USG abdomen, liver biopsy performed
Day 4-7	Started UDCA, antihistamines, cholestyramine, and other supportive therapy
Day 10-13	Worsening bilirubin and INR, GGT 312 IU/L, biopsy revealed intracanalicular and patchy intracellular cholestasis
Day 15	After ruling out common acquired causes and worsening cholestasis, several genetic disorders like BRIC, PFIC, and Alagille syndrome were considered. Hence, WES was ordered
1 month	WES confirmed heterozygous pathogenic JAG1 mutation (c.765C>A, p.Cys255*); multiorgan screening was normal
2-4 months	Patient was on maximum doses of UDCA and also continued cholestyramine along with supportive therapy
4 months	Resolution of jaundice and pruritus, LFT normalized
Ongoing	Maintenance UDCA and HCC surveillance and genetic counseling

On examination, the patient was conscious and well oriented to time, place, and person. Vital signs were stable. General physical examination revealed icterus with excoriations secondary to pruritus. However, there were no stigmata of chronic liver disease or any characteristic facial dysmorphic features. Abdominal examination revealed no hepatosplenomegaly. Table 2 depicts the thorough laboratory evaluation along with liver biopsy findings.

**Table 2:** Laboratory investigations of the patient. ALT: alanine aminotransferase; AST: aspartate aminotransferase; ALP: alkaline phosphatase; GGT: gamma-glutamyl transferase; INR: international normalized ratio; CECT: contrast-enhanced computed tomography; HAV: hepatitis A virus; HBV: hepatitis B virus; HCV: hepatitis C virus; HEV: hepatitis E virus; JAG1: Jagged canonical Notch ligand 1

Parameters	Patient values	Reference range
Total bilirubin	7.3 mg/dL (on presentation) → 9.1 mg/dL → <1.2 mg/dL (at 4 months)	0.1-1.2 mg/dL
Direct bilirubin	6.4 mg/dL (on presentation) → 8 mg/dL → 0.3 mg/dL (at 4 months)	0-0.3 mg/dL
ALT	68 U/L	7-56 U/L
AST	27 U/L	8-48 U/L
ALP	167 IU/L	44-147 IU/L
GGT	312 IU/L → 45 IU/L (at 4 months)	9-48 IU/L
INR	1.6	0.8-1.2
Viral hepatitis serologies (HAV, HBV, HCV, HEV)	Negative	-
Autoimmune markers	Negative	-
Serum ceruloplasmin	Normal	-
FibroScan	10.2 kPa	<7 kPa
CECT abdomen	Mild intrahepatic duct dilatation	-
Liver biopsy	Prominent intracanalicular and patchy intracellular cholestasis (no evidence of bile duct paucity)	-
Whole exome sequencing	Heterozygous pathogenic JAG1 variant (c.765C>A, p.Cys255*)	-
Alpha-fetoprotein	<10 ng/mL	Normal

The patient was thoroughly investigated as demonstrated in the table above to rule out common acquired etiologies. Slit-lamp examination showed no Kayser-Fleischer ring, and upper gastrointestinal endoscopy showed no evidence of portal hypertension. At this point, uncommon causes of recurrent cholestasis such as benign recurrent intrahepatic cholestasis (BRIC) and progressive familial intrahepatic cholestasis (PFIC) were kept as differential diagnoses. However, the liver biopsy showed intracanalicular cholestasis with no evidence of bile duct paucity. Genomic evaluation was then considered to rule out the differential diagnoses, as liver biopsy findings were not definitive. Whole exome sequencing (WES) was performed and confirmed a heterozygous pathogenic JAG1 variant (c.765C>A, p.Cys255*). This variant introduces a premature stop codon, resulting in a truncated protein. The variant was classified as pathogenic according to the American College of Medical Genetics and Genomics (ACMG)/Association for Molecular Pathology (AMP) guidelines, which was also consistent with the diagnosis of Alagille syndrome. Multisystem screening was done to look for organ-related anomalies like spine X-ray, MRI, angiography, renal ultrasound, and 2D echocardiography, which were unremarkable. Slit-lamp examination showed no posterior embryotoxon.

Management included ursodeoxycholic acid (UDCA) 15 mg/kg/day in divided doses, cholestyramine 4 g every 12 h, along with antihistamines and fat-soluble vitamin supplementation. The patient showed clinical and biochemical improvement while receiving UDCA and supportive therapy. Of note, a previous episode had also resolved spontaneously, so a direct causal effect of UDCA cannot be definitively established. The patient was then discharged on maintenance doses of UDCA and supportive medical therapy. Genetic counseling was provided regarding inheritance risk for offspring, and genetic testing of offspring was advised. At four-month follow-up, the patient remains asymptomatic on maintenance ursodeoxycholic acid (UDCA) with regular surveillance of liver function tests, imaging, and hepatocellular carcinoma (HCC) screening.

## Discussion

Alagille syndrome is a complex inherited genetic disorder with a wide spectrum of clinical variability due to mutations in the JAG1 or NOTCH2 gene [3,6]. The initial diagnostic criteria described Alagille syndrome predominantly as a hepatic disease characterized by bile duct paucity (duct-to-portal tract ratio of less than 0.5) on liver biopsy along with other salient features including cholestasis, cardiac defects (e.g., peripheral pulmonary stenosis), skeletal abnormalities (butterfly vertebrae), ocular malformations (posterior embryotoxon), or facial dysmorphism (such as hypertelorism, deep-set eyes, prominent chin) [2].Traditionally, more than three major clinical features out of five along with bile duct paucity were required for the diagnosis of classical Alagille syndrome [7]. Renal anomalies (renal dysplasia) and vascular malformations in the head and neck were also later incorporated into the diagnostic criteria [8,9]. However, with advances in molecular diagnostics, the criteria were revised by Turnpenny and Ellard and Kamath et al. to include one or more characteristic clinical features and JAG1 or NOTCH2 mutation [1,10].Even in the absence of overt clinical manifestations, individuals with a positive family history and defects should be evaluated for Alagille syndrome. Conversely, some patients with confirmed Alagille syndrome may not have a positive family history due to incomplete penetrance and failure to recognize symptoms in the family members. Therefore, a negative family history cannot rule out the diagnosis of Alagille syndrome without proper genetic and clinical evaluation of parents [4].In the present case, the patient's sibling had a history of jaundice but was deceased, and limited details were available; thus, the family history could not be reliably confirmed or used to establish a clear inheritance pattern.

Based on the recent clinical diagnostic criteria, our patient exhibited cholestasis, as evidenced by markedly elevated gamma-glutamyl transferase (GGT), total bilirubin of 7.3 mg/dL (direct>indirect), and raised alkaline phosphatase (ALP), along with a pathogenic JAG1 mutation identified by whole exome sequencing (WES), confirming the diagnosis of Alagille syndrome. Although bile duct paucity is the histological hallmark of Alagille syndrome, it is not always present, particularly in adult-onset or milder phenotypes. The absence of classical liver biopsy findings in our patient, despite a confirmed pathogenic variant, is consistent with the known phenotypic variability of the disease and supports the increasing role of molecular testing in atypical cases [3]. However, it remains an essential component of clinical diagnosis when genetic testing is unavailable in a timely manner or to differentiate Alagille syndrome from other causes of intrahepatic cholestasis [11].

The primary aim is to focus mainly on symptomatic relief to slow down the disease process. Choleretic agents like ursodeoxycholic acid (UDCA) help alleviate pruritus, often in combination with cholestyramine, rifampin, or naltrexone. Multimodal therapy, including optimized nutrition and fat-soluble vitamin supplementation, is required. Novel therapies, such as ileal bile acid transport inhibitors, such as maralixibat and odevixibat, which are FDA-approved for management of cholestatic pruritus in Alagille syndrome, offer additional options [12]. Liver transplantation is often recommended in patients progressing to end-stage liver disease, with a five-year survival rate of approximately 80% [13]. External biliary diversion has also been shown to be useful in select cases prior to transplantation [14]. Given the increased risk of hepatocellular carcinoma (HCC) in adult patients with Alagille syndrome, including those with milder hepatic phenotype, regular surveillance and cancer screening are advised for early detection and management of at-risk patients [15].

This case illustrates the diagnostic odyssey of a patient with Alagille syndrome who presented in adulthood, in which recurrent cholestasis was initially misattributed to acquired causes. However, timely genetic testing and prompt management with choleretic agents like ursodeoxycholic acid (UDCA) led to confirmation of the diagnosis and successful clinical resolution.

## Conclusions

Alagille syndrome should be included in the differential diagnosis of recurrent intrahepatic cholestasis in adults, even in the absence of any dysmorphic or other characteristic manifestations. Unexplained cholestasis in adults frequently masks underlying genetic etiologies behind more common acquired causes. This case highlights the significance of incorporating molecular diagnostic testing like whole exome sequencing (WES) to facilitate definitive diagnosis and reduce diagnostic delays in such rare disorders. While this single case supports the diagnostic value of genetic evaluation in selected patients, it does not establish that whole exome sequencing should be performed routinely in all patients with unexplained cholestasis. Rather, genetic testing can complement clinical, biochemical, imaging, and histopathological assessment when an inherited disorder is suspected.

## References

[1] Turnpenny PD, Ellard S (2012). Alagille syndrome: pathogenesis, diagnosis and management. Eur J Hum Genet.

[2] Alagille D, Odièvre M, Gautier M, Dommergues JP (1975). Hepatic ductular hypoplasia associated with characteristic facies, vertebral malformations, retarded physical, mental, and sexual development, and cardiac murmur. J Pediatr.

[3] Li L, Krantz ID, Deng Y (1997). Alagille syndrome is caused by mutations in human Jagged1, which encodes a ligand for Notch1. Nat Genet.

[4] Spinner NB, Loomes KM, Krantz ID, Gilbert MA (2000). Alagille syndrome. GeneReviews [Internet].

[5] Li J, Wu H, Chen S, Pang J, Wang H, Li X, Gan W (2023). Clinical and genetic characteristics of Alagille syndrome in adults. J Clin Transl Hepatol.

[6] Saleh M, Kamath BM, Chitayat D (2016). Alagille syndrome: clinical perspectives. Appl Clin Genet.

[7] Alagille D, Estrada A, Hadchouel M, Gautier M, Odièvre M, Dommergues JP (1987). Syndromic paucity of interlobular bile ducts (Alagille syndrome or arteriohepatic dysplasia): review of 80 cases. J Pediatr.

[8] Krantz ID, Colliton RP, Genin A, Rand EB, Li L, Piccoli DA, Spinner NB (1998). Spectrum and frequency of Jagged1 (JAG1) mutations in Alagille syndrome patients and their families. Am J Hum Genet.

[9] Kamath BM, Podkameni G, Hutchinson AL (2012). Renal anomalies in Alagille syndrome: a disease-defining feature. Am J Med Genet A.

[10] Kamath BM, Ye W, Goodrich NP (2020). Outcomes of childhood cholestasis in Alagille syndrome: results of a multicenter observational study. Hepatol Commun.

[11] Ayoub MD, Bakhsh AA, Vandriel SM, Keitel V, Kamath BM (2023). Management of adults with Alagille syndrome. Hepatol Int.

[12] Shneider BL, Spino CA, Kamath BM (2022). Impact of long‐term administration of maralixibat on children with cholestasis secondary to Alagille syndrome. Hepatol Commun.

[13] Kasahara M, Kiuchi T, Inomata Y (2003). Living-related liver transplantation for Alagille syndrome. Transplantation.

[14] Mattei P, von Allmen D, Piccoli D, Rand E (2006). Relief of intractable pruritus in Alagille syndrome by partial external biliary diversion. J Pediatr Surg.

[15] Schindler EA, Gilbert MA, Piccoli DA, Spinner NB, Krantz ID, Loomes KM (2021). Alagille syndrome and risk for hepatocellular carcinoma: need for increased surveillance in adults with mild liver phenotypes. Am J Med Genet A.

